# Supplementary figures and images for: Natural scene statistics predict how humans pool information across space in surface tilt estimation
Source: PLoS Comput Biol. 2020 Jun 24;16(6):e1007947. doi: 10.1371/journal.pcbi.1007947 (PMC7340327; doi:10.1371/journal.pcbi.1007947)

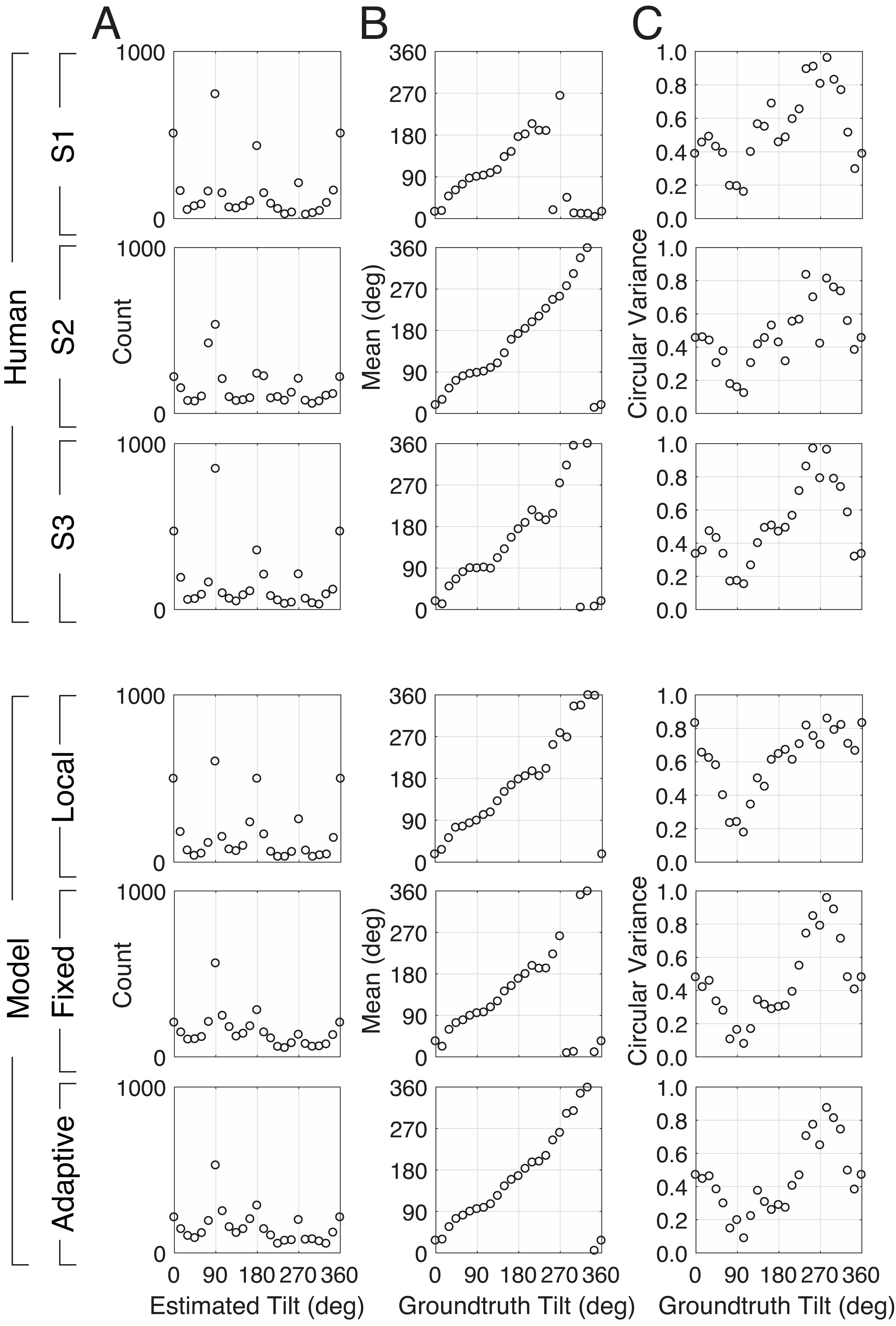

Supplement: S1 Fig — A Histogram of raw responses. B Mean of tilt estimates as a function of groundtruth tilt (binned in 24 bins). C Variance of tilt estimates as a function of groundtruth tilt. Data from the fixed circular pooling model corresponds to the best pooling area (i.e., 1º diameter). Data from the adaptive elliptical model corresponds to the adaptive model with the best average pooling area (i.e., 1º average equivalent diameter). The variances of the human tilt estimates are substantially more similar to the variances of the tilt estimates from the global pooling models (fixed & adaptive) than from the local model. (TIF) [file pcbi.1007947.s001.tif]

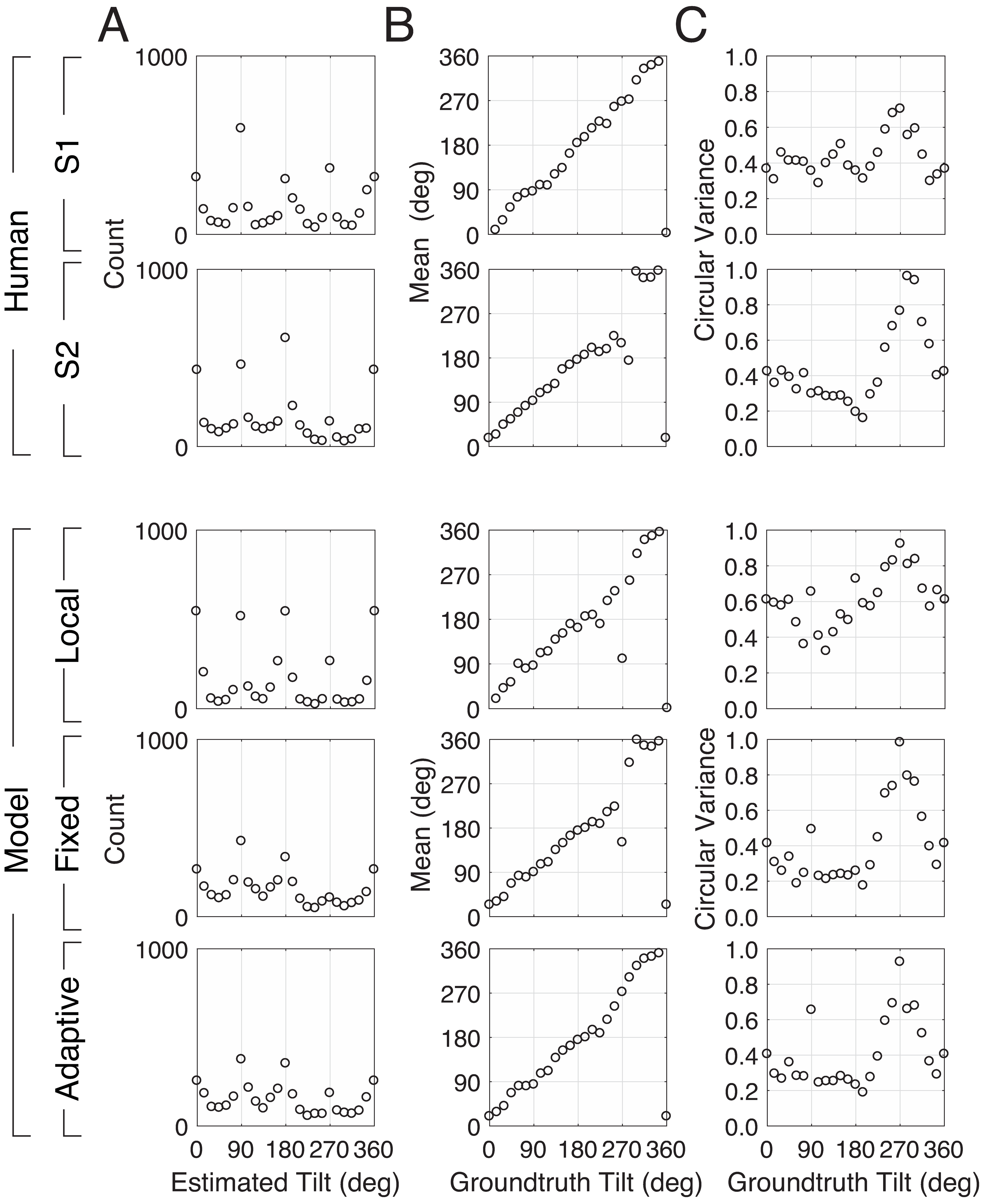

Supplement: S2 Fig — A Histogram of raw responses. B Mean of tilt estimates as a function of groundtruth tilt (binned in 24 bins). C Variance of tilt estimates as a function of groundtruth tilt. Data from the fixed circular pooling model corresponds to the best pooling area (i.e., 1º diameter). Data from the adaptive elliptical model corresponds to the adaptive model with the best average pooling area (i.e., 1º average equivalent diameter). The variances of the human tilt estimates are substantially more similar to the variances of the tilt estimates from the global pooling models (fixed & adaptive) than from the local model. The variances of the human and model tilt estimates in Exp 2 exhibit substantially different patterns than in Exp 1. The differences are due to the different sets of natural stimuli that were presented during the experiment. (TIF) [file pcbi.1007947.s002.tif]

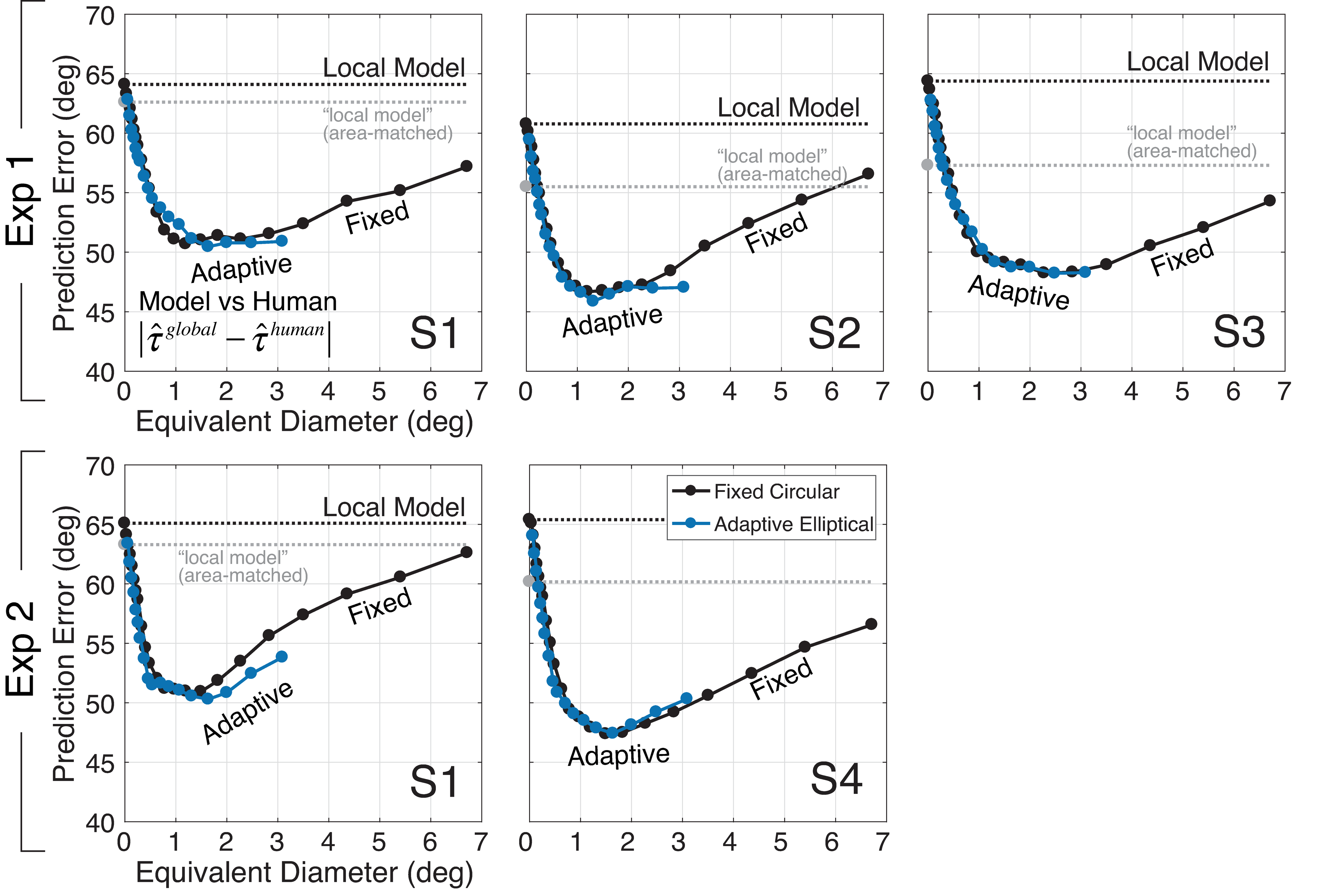

Supplement: S3 Fig — Prediction error is shown for the fixed circular pooling model (black), the adaptive elliptical pooling model (blue). The top and bottom rows indicate results from Exp 1 and Exp 2, respectively. The pooling region that minimizes prediction error for all models and all human observers (except observer S3) corresponds to an equivalent pooling diameter between 1º and 2º. The black dashed line indicates the prediction error for the local model. The gray dashed line indicates the prediction error for a “local” model that computes the image cues from an area matched to that implicitly used by the best global model. (TIF) [file pcbi.1007947.s003.tif]

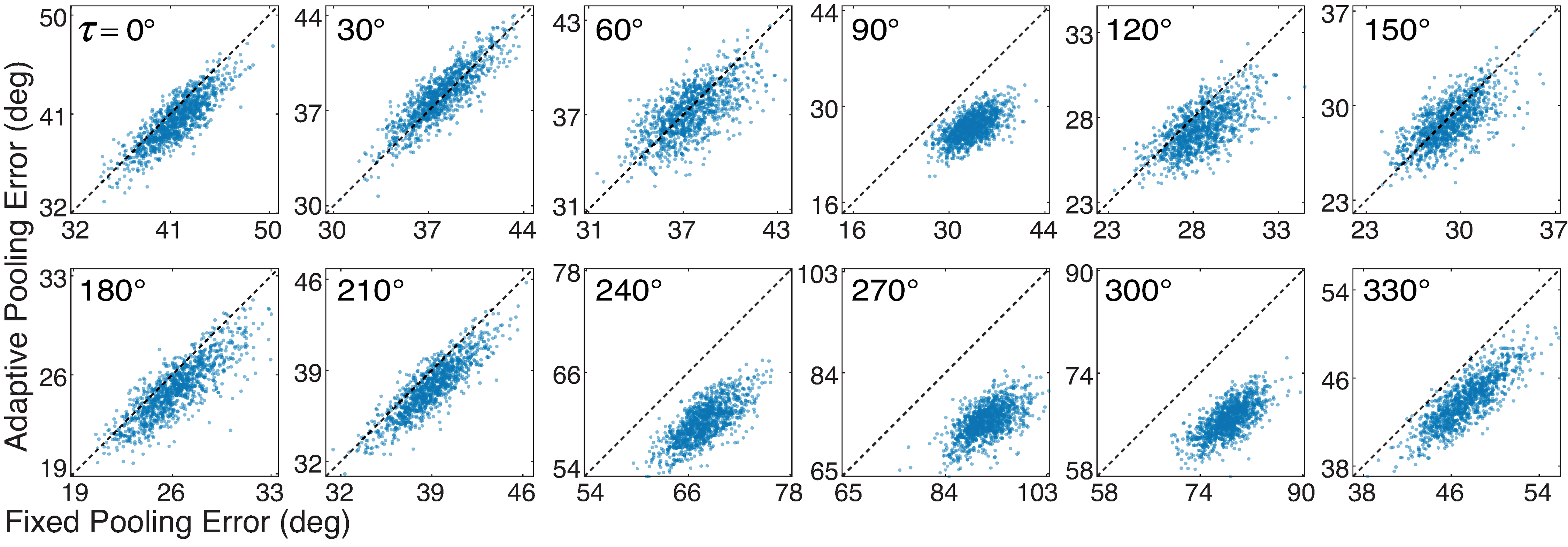

Supplement: S4 Fig — Each point represents the mean estimation error in a randomly sampled stimulus set across stimuli at a given groundtruth tilt. Estimation error with fixed circular pooling is plotted against estimation error with adaptive elliptical pooling. Computing the prediction errors on matched stimulus sets isolates the impact of the model, and prevents stimulus variability from unduly affecting the results. The fact that the majority of points lie below the dashed unity line, indicating that adaptive elliptical pooling outperforms fixed circular pooling for the task of estimating groundtruth tilt in natural scenes. (TIF) [file pcbi.1007947.s004.tif]

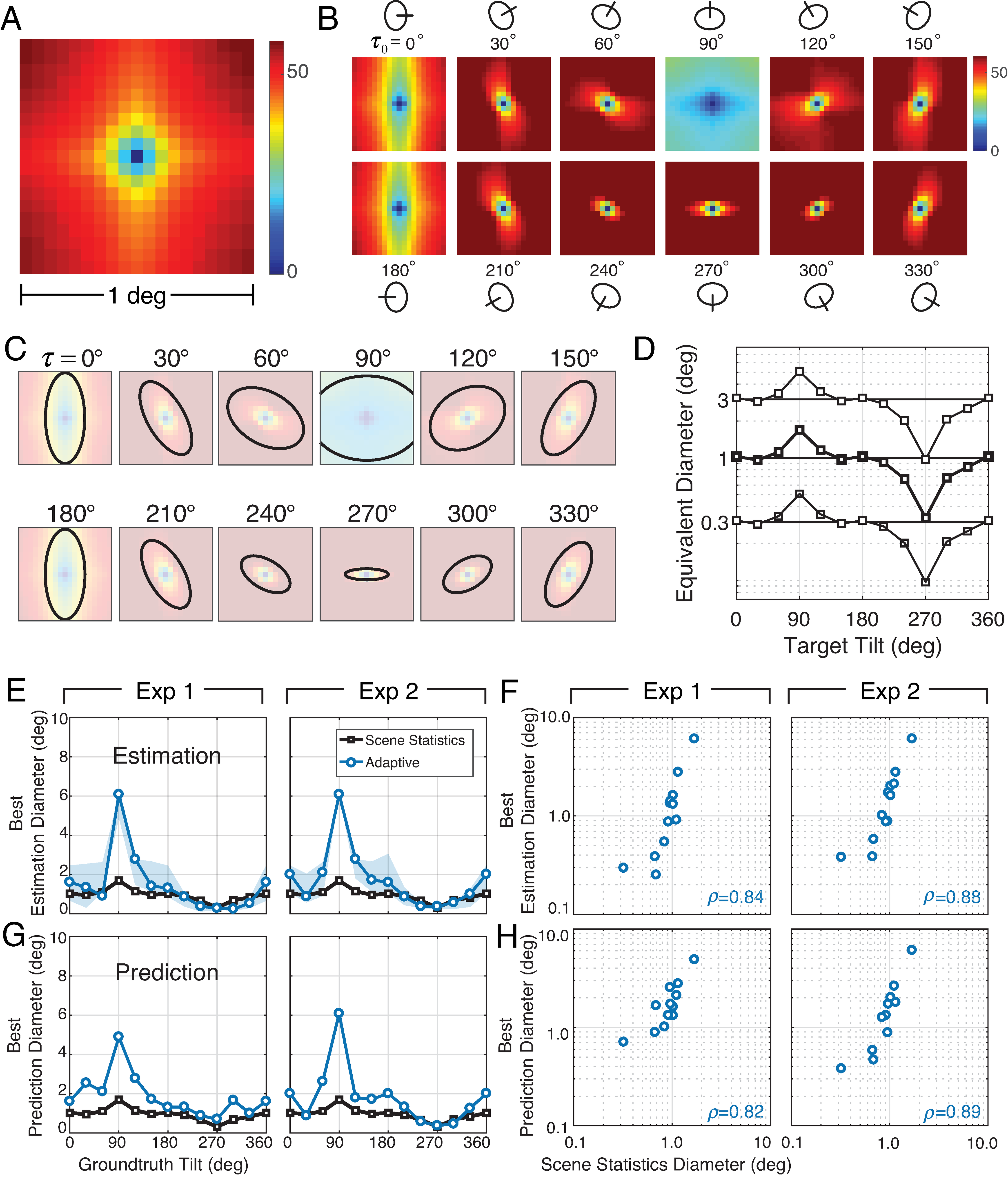

Supplement: S5 Fig — A Spatial statistics of tilt in natural scenes over a 1deg area. Mean absolute tilt difference as a function of spatial location relative to a target location. B Mean absolute tilt difference conditioned on the groundtruth tilt at the target location. C Fits to the scene statistics in B. D Equivalent diameters of the fits to the scene statistics in C. E Adaptive pooling regions predicted by natural scene statistics predict the pooling regions that maximize performance at each groundtruth tilt. Equivalent pooling diameters fit to the natural scene statistics (black) and equivalent pooling diameters that minimize estimation error (blue), plotted as a function of groundtruth tilt. The left and right columns represent data from Exp 1 and Exp 2, respectively. F Best estimation diameters are correlated with the diameters fit to the natural scene statistics. G Equivalent pooling diameter fit to the natural scene statistics and equivalent pooling diameters that minimize prediction error, plotted as a function of groundtruth tilt. H Best prediction diameters are correlated with the diameters fit to the natural scene statistics. (TIF) [file pcbi.1007947.s005.tif]
