# Supplementary material for: Natural scene statistics predict how humans pool information across space in surface tilt estimation
Source: PLoS Comput Biol. 2020 Jun 24;16(6):e1007947. doi: 10.1371/journal.pcbi.1007947 (PMC7340327; doi:10.1371/journal.pcbi.1007947)
Supplement: Attachment — Submitted filename: KimBurge_PLoSCB_2019_rsp2rvw.docx [file pcbi.1007947.s006.docx]

Dear Editors and Reviewers,

We appreciate the time and effort that the editors and reviewers took to provide careful and thoughtful feedback on our paper. We believe the changes that we made to the manuscript in response to the reviewer’s comments have substantially strengthened the paper. We hope the revised manuscript is judged to be suitable for publication. Please find below a point-by-point response to each reviewer’s comment. Reviewer comments are in italic and bold type. Our responses are in regular type.

Thank you for your consideration.

Sincerely,

Seha Kim, PhD

Johannes Burge, PhD

*****************************************************************************************

Reviewer #1: Review of "Natural scene statistics predict how humans pool information across space in surface tilt estimation"
Reviewer: Michael Landy

This paper describes experimental data on estimation of surface tilt from patches of stereo images from a natural-image database that is coupled with range data, so that ground-truth tilt is known. They find that adding spatial context (local estimates from surrounding pixels) improves the performance of their computational observer and also improves the match of predicted responses with human responses, suggesting that human tilt estimation also uses surrounding context to improve estimates and make them more robust.

This is a nice paper, clearly written and with clear results.

Thanks for these remarks.

I suppose my main overall reactions are twofold. First, This is a follow-up to their previous paper (Ref. 16) and yet this relationship is only made clear in a single sentence (bottom of p. 6). This should be discussed in the introduction, so that it is made clear what the contribution of this paper is above and beyond the previous paper.

Apologies. This was not our intention. Our earliest drafts of the paper spent too much time reviewing our last paper before we got to the point of the current paper. In our efforts to streamline the manuscript during our internal revision process, we over corrected. We now make the relationship with our previous paper clear in the introduction.

Also, as I'll mention below, there are spots where knowledge of the methods of the previous paper are required to make sense of the current one, and this one should be able to be read on its own.

We added more details about the previous paper in various places as appropriate (see below).

Second, there really isn't a clear description of how good the improvement of prediction with context is, compared to how good an improvement one might expect. It's a decrease in prediction error of 10 deg or so, but is there an independent measure of estimation variance, so that one can get an absolute sense of whether that's all the improvement any model can manage or, contrarily, whether that's actually fairly weak evidence?

Good point. We do not have a wholly satisfying response, but we do have the results of one new analysis to present. We constructed a toy model that outputs a tilt ‘estimate’ that is equal to the circular mean of the human estimates to each stimulus. This model minimizes the prediction error of the average human subject. This model provides a best-case-scenario in terms of its ability to minimize mean prediction error (c.f. Fig. 8), but it is a wholly implausible working model of tilt estimation because it operates only post hoc on the human responses and not on the image data. Nevertheless, it provides a potentially useful benchmark for comparison. Across all stimuli, this model improves prediction error by 39deg over the local model. (Note: the global model improves prediction error over the local model by 15deg). The model also improves estimation error by ~13deg over the local model. (Note: the global model improves estimation error by 14deg). We have added the discussion of these points to the manuscript. We hope that this new analysis at least partially addresses the reviewer’s question.

Specifics (page/para/line):

4/3: The slant/tilt of a surface is really a multi-scale phenomenon, at least for surfaces that have local 3-d texture. Thus, the estimate from the database will be a function of the resolution of the measurements (in surface coordinates, i.e., the scale will also be affected by distance to the camera). I don't know whether that's worth a mention/discussion.

Very good point. Before receiving your review, we actually started drafted a

discussion section paragraph that discusses exactly this issue. It is titled ‘The definition of groundtruth tilt’ and is now included in the Discussion.

Overall: You never really show any raw data (i.e., the distributions of estimates conditioned on the ground-truth value). These are presumably in the other paper, but a reader might be curious about whether there are a lot of tilt-sign errors and, as mentioned above, the amount of variance.

We now show the estimate counts, means, and variances for each human observer in the new Supplementary Figures S1 and S2.

Fig. 3: I have no idea what a "mouse-controlled graphical probe" is, and Fig. 3A certainly doesn't help! ;^)

We now describe what a mouse-controlled graphical probe is in more detail in both the main text and in the relevant figure caption.

Eq, 2: This is elaborate, but isn't this just going to turn out to be the sign of the signed disparity cue (i.e., expectation over the database isn't going to change that estimate)?

Close, but not quite. It is the sign of the signed estimate based only on the signed disparity cue. This is not quite the same thing as the sign of the disparity cue. Please also note that in Eq. 2, there was a typographical error that we have now fixed.

10/1/4: "the human estimate": You never say what this is (i.e., presumably a circular average across subjects, I guess).

Good point. We now specify that we computed the prediction error as the absolute circular distance between the model estimate and the average human estimate. In practice, we first computed the absolute circular distance between the model estimate and the human estimate on each trial and then took the average across observers and stimuli. We added these details in the main text and Methods section.

Fig. 9D: The 90 deg data show an interesting phenomenon that isn't discussed (it's also in Fig. S2): fixed closer to adaptive for local-tilt at 90 deg but farther at 270. I'm not sure what, if anything, that means. But, it makes me wonder about the lack of any discussion of tilt priors (preference for cardinals, preference for ground plane over ceiling plane in this case). Sure, experimentally you impose a flat prior, but that doesn't mean the observers ignored their priors.

The reviewer is quite right that we should discuss the tilt prior. We imposed a flat prior over tilt in the experimental stimulus sets, but we did not assume that the observers (model or human) ignored the tilt prior in natural scenes. The model estimates are clearly consistent with the action of a cardinal tilt prior, and the human estimates are too. We have included a plot of the distribution of tilts in natural scenes (i.e. our database) in Fig. 2C, and described the tilt prior both in the scene statistics section accompanying this figure and how it impacts the model, and how it influences human estimates counts, means, and variances in the new Supplementary Figures S1 and S2.

We also find the phenomenon in Fig. 9D and Fig. S2 interesting, but we are not quite sure what to make of it.

Many places: You spend a bunch of the paper comparing a model that chooses the pooling region/weights based on the ground-truth local tilt vs. the local estimate. Yet, you never once mention that the former is nonsensical as a model of human (or any) observer behavior, since ground truth is unknown to the observer. I don't really get the point of even including the ground-truth version of the model, given its logical circularity.

We actually had made the point that the model is not plausible, but perhaps not forcefully enough. But we agree with the reviewer. We have removed the discussion of the groundtruth version of the model from the manuscript.

Fig. 11B/D: These are correlated, but not slope 1, not identity line, and this is log-log, so the suggestion is that the two don't match and are related by a power function. Huh?

Yes. It is a little surprising. We gained some intuition by examining Fig. 11AC. The fit to the scene statistics (black curve) clearly follows the same pattern that governs the human data (colored curves). A simple scale factor on the natural scene stats fits (i.e. linear relationship) does not provide a great match to the human data, but raising the scene stats fits to a positive power larger than 1.0 (i.e. simple non-linear relationship) provides a pretty good match. We were a bit surprised by this finding as well, and do not pretend to fully understand it. But the data is how it is. Note that this same relationship holds approximately in Supplementary Fig. S5.

15/1/14: Fig. 11B -> Fig. 11C

Fixed.

19/1: How many trials per block?

150 trials per block. This information has now been added to the manuscript.

19/2/2: A four-quadrant arctan can't be a function of the ratio, but needs the individual values (as it does in Matlab!).

Fixed*.*

19/2/5: Are the partial derivatives simply first differences in x and y (of neighboring pixels) or what?

The gradients were computed at a particular spatial scale. We convolved the range data and cue images with first-order Gaussian derivative kernels in x and y; that is, the first derivatives of a Gaussian kernel in x and y. The spatial scale was determined by the standard deviation of the kernel. The kernels were computed analytically. This is equivalent to first convolving with a Gaussian and then taking the partial derivatives in x and y. The equivalence is due to the fact that convolution and differentiation are both linear operations, so the derivative of a convolution with a kernel is the same as convolving with the derivative of the kernel. Hope this helps.

19/3/7: "the derivative operator": again, I'm not sure exactly what precise computation is being referenced by this.

See above.

19/4/1: "the mean tilt difference": Don't you mean the mean ABSOLUTE tilt difference here?

Yes. Fixed.

20/2/4-6: Why are we talking about a "posterior" when we've never talked about a prior? Are you considering the database to be a prior? Or the subsampled one designed to be flat over tilt?

Thanks for pointing out this oversight. Please see above. We now state that that the model implicitly assumes a prior over tilt that is consistent with the distribution of tilts in the database.

Eqs. 7 & 8: First, since you are computing the arg(), the 1/N is not needed.

We know. We thought that, for some readers, it might be nice to see the familiar 1/N in the expression for the average. Now we have removed it.

Second, you should clarify that you aren't conditioning on the exact values of the three cues (c_u), but rather on the bin (of the 64x64x64 binning, that you never clearly describe in this paper, and should).

Fixed. We have added the description in the Methods section.

Reviewer #2: This is an elegant and detailed study of the adaptive pooling of local cues to surface tilt across stereoscopic, luminance and texture cues. The authors use a large, high-fidelity dataset of co-registered image and depth data to measure the spatial statistics of surface orientation in natural scenes, and use this to determine the optimal pooling of signals to improve tilt estimation. They find that the optimal pooling patterns are anisotropic, such that for a given tilt at a given image location, estimation can be improved by pooling signals over elliptical neighbourhoods, favouring directions aligned with the projected tangent plane. They then compare the predictions of the optimal model with human tilt estimation performance, to test the hypothesis that the human visual system also optimally pools local tilt estimates. They consider both isotropic and anisotropic pooling models. The findings show convincingly that the human visual system does pool local signals, and the analyses in Figures 11 and 12 suggest that the visual system does so in an adaptive way that is consistent with the statistics of natural images (at least in terms of pooling region size).

Thanks for these remarks.

I don’t have major comments, but I do have a number of minor issues and suggestions. The authors should say more about the difference between Experiments 1 and 2. This includes on p.6: the main text should indicate more clearly that there are two separate psychophysical experiments, and should briefly explain the difference between them. Otherwise, references to Experiments 1 and 2 are confusing throughout the rest of the MS.

Good point. We have addressed this issue in a paragraph that has been added to the ‘Psychophysical experiment’.

More importantly, a subsection should be added to the discussion section, discussing the difference in prediction quality for the two experiments. Is the problem with higher slants (Experiment 1) the presence of artifacts in the image set? Or is the fact that higher slants tend to be associated with depth discontinuities within the pooling region? Were the results of Experiment 1 evaluated before deciding to run Experiment 2? Some rationale and discussion would be helpful.

As requested, we have added a new sentence to the Introduction and a new section to the Discussion titled ‘Two psychophysical experiments’. We hope this addresses the reviewer’s concern.

It would also be helpful to be given stronger intuitions about how local estimates are affected by estimates that come from outside the pooling region. Each pixel within the pooling region for a given local estimate is itself adjusted by its own pooling region. Does this ‘cascading’ extend to the whole image? If not, why not? How quickly does the effect dilute as a function of distance outside the pooling region? The authors could add an analysis to quantify this (it should not be so difficult to do: values can be clamped to the local estimates vs. all values modified by their own pooling regions).

Local estimates were based on gradients of the image properties in a small fixed area of the image. Local estimates that were separated by more than this fixed area had no common inputs (i.e. image pixels). Very nearby local estimates may be correlated, but this is because very nearby local estimates share input image pixels. If the reviewer is asking whether the global estimate at the target location was obtained by pooling neighboring global (instead of local) estimates, this is not what we did. On average, the size of the best global pooling region was substantially larger than the image area that contributed to each local estimate. To show that the benefit of global pooling is not simply due to using information from a larger area of the image, we examined performance for a larger area-matched ‘local’ model (i.e. no global pooling) that explicitly used pixel data from the same image region implicitly used by the best global pooling model. The large area-matched ‘local’ model underperforms the best global model in terms of both estimation and prediction errors (see below). Hopefully, this response and our additional analysis have helped.

It would also be useful to understand the relative contribution of the three different cues in the local estimates. How far off is the disparity estimate from the nominal ground truth? Does this boil down to basically a study of how to compute optimal derivatives of depth-from-stereo estimates? A few sentences on this would be useful.

Good question. We conducted this analysis in a previous paper, but we agree that it is useful to mention the results here. Briefly, when the other two cues differ from disparity and approximately agree with each other, the model estimate differs considerably from the estimate that would be obtained from disparity alone (see Fig. 11A from Burge et al., 2016 below). When the other two cues substantially differ from each other, they change the disparity-alone estimate by a small amount (Fig. 11E). These adjustments to the disparity-alone estimate are all in the service of improving groundtruth estimation performance. We now mention these results in the manuscript.

Why are the estimated and prediction errors so high? 60 degrees error for the local estimates seems quite large, especially considering that chance is 90 degrees. That means that tilt estimates are closer to random than to the ground truth. Or have I misunderstood something?

Estimating tilt from very local image cues is just hard, and especially so for some stimuli. Although the model and human estimates tend to be clustered around the correct tilt (Kim & Burge, 2018), the distributions of the model and human estimates across stimuli at each groundtruth tilt tend to have heavy tails. As a result, both the model and humans produce a non-negligible number of very large errors. These large errors are important contributors to the high estimation and prediction errors. Please also see our response to Reviewer 1 above and the new Supplementary Figures S1 and S2 that show the estimate counts, means, and variances.

Figure 2D: an indication of the marginal frequency of the different tilts would be helpful. For example, 90 deg is presumably significantly more common than others.

Yes. We now plot the distribution of tilts in the natural scene database in Fig 2C.

An interesting control condition for the experiment would be to rotate the image patches by various amounts so that their local statistics would be the same, but their relationship to the global statistics of tilts in the real world would be changed. Would the best pooling model rotate with the patches or not? This would provide insights into the cues the visual system uses to adapt the pooling region: is it just a prior based on the local tilt estimate, or is it based on recognizing the image content in some way? I don’t think it is necessary to perform this experiment for the paper to be publishable, but it might be interesting to mention it in the discussion section, or, ideally do a small control condition with a smaller number of image patches.

Interesting idea. We rotated the patches in Experiments 1 and 2 and obtained the local tilt estimates. In general, the local tilt estimate is rotated with the patch but with a weaker influence of the prior over tilt. The distribution of the local tilt estimates is a little more similar to a uniform distribution than the distribution of model estimates in the manuscript (the left plot). That said, the mean local tilt estimates as a function of the rotated groundtruth (i.e., the original groundtruth +45 deg) still shows some cardinal biases (the center plot), an effect that is likely to be due to the prior. The pattern of variance is altered noticeably. This implies that the biases towards the cardinal angles are due both to the prior and to the local image properties.

Typos etc:
p. 8 ‘properties of tilt natural scenes’ -> ‘properties of tilt in natural scenes’

Fixed.

Figures 7 and 8: Why is the mean estimate so close to the lower bound of the confidence intervals in Experiment 1 and so close to the top in Experiment 2 (at least above 2deg)?

We presume the reviewer is asking about the two plots in Fig. 7 not the plots in Fig. 8. We are honestly not sure. We have confirmed that the plots are correct, so it must be for large pooling region sizes, it just so happened that our particular datasets were somewhat atypical. It just happened to fall outside the 95% confidence interval in those particular conditions.

p. 15: ‘should be best accounted for similarly sized pooling regions’. There is a missing ‘by’.

Fixed.

p. 16: ‘If humans use the pooling regions that yield the most accurate performance, humans are doing the right thing’. Perhaps a less colloquial formulation can be found?

We replaced ‘doing the right thing’ with ‘appropriately using the available information’.
